# Does a Change from Whole to Powdered Food (*Artemia franciscana* eggs) Increase Oviposition in the Ladybird *Coleomegilla maculata*?

**DOI:** 10.3390/insects6040815

**Published:** 2015-09-28

**Authors:** Eric W. Riddick, Zhixin Wu

**Affiliations:** National Biological Control Laboratory, Jamie Whitten Delta States Research Center, ARS-USDA, Stoneville, MS 38776, USA; E-Mail: zhixin.wu@ars.usda.gov

**Keywords:** Artemiidae, Coccinellidae, biological control, lady beetle, lipids, predator

## Abstract

The limited availability of alternative foods to replace natural prey hinders cost-effective mass production of ladybird beetles for augmentative biological control. We compared the effects of powdered *vs.* whole *Artemia franciscana* (*A. franciscana*) (brine shrimp) eggs with or without a dietary supplement on development and reproduction of *Coleomegilla maculata* (*C. maculata*) (Coleoptera: Coccinellidae). We tested the hypotheses that (1) powdered *A*. *franciscana* eggs are more suitable than whole eggs; and (2) palmitic acid, a common fatty acid in natural prey, *i.e*., aphids, is an effective dietary supplement. Development time, pre-imaginal survival, sex ratio, and body weight of adults did not differ significantly amongst individuals fed powdered *vs*. whole eggs, with or without 5% palmitic acid. Significantly more oviposition occurred when females were fed powdered eggs than whole eggs and powdered eggs with 5% palmitic acid than whole eggs with or without 5% palmitic acid. A weak functional relationship was found between pre-oviposition time and total oviposition by females fed powdered eggs with 5% palmitic acid; pre-oviposition time decreased as oviposition increased. Food treatments had no significant differential effect on progeny (egg) hatch rate. In conclusion, a simple change in *A*. *franciscana* egg texture and particle size (*i.e*., blending whole eggs into a dust-like powder) increases oviposition in *C*. *maculata*. Supplementing powdered eggs with 5% palmitic acid might accelerate oogenesis (egg maturation) in some females.

## 1. Introduction

*Coleomegilla maculata* (*C. maculata*) DeGeer (Coleoptera: Coccinellidae) is a native ladybird beetle found in natural and managed ecosystems in North, Central, and South America [1,2,3,4]. It is polyphagous, consuming eggs and young larval stages of soft-bodied insects and mites, with a preference for aphids [5,6,7] and plant pollen, especially during periods of prey shortage [8,9]. There is interest in using this ladybird beetle for conservation biological control of pests in field crops [10,11,12], for testing non-target effects of plant toxins [13], and for augmentative biological control of plant pests in protected culture, *i.e*., high tunnels, greenhouses, plantscapes and interiorscapes [14,15,16].

A challenge to mass-producing coccinellids, such as *C*. *maculata*, is the availability of inexpensive, nutritious, alternative foods or artificial diets to ensure development, growth, and reproduction. Since natural prey (such as most aphid species) often require live host plants as food, alternative foods or artificial diets are needed to cut costs associated with rearing *C*. *maculata*. The best solution would involve using an artificial diet, devoid of any insect (or arthropod) components, which permit multigenerational rearing of healthy individuals [17,18]. No such stand-alone, arthropod-free artificial diet is currently available, to our knowledge, but research has been ongoing [19,20,21,22]. One alternative (factitious) food for rearing *C*. *maculata* and other aphidophagous coccinellids such as *Harmonia axyridis* (Pallas) and *Adalia bipunctata* L., are eggs of the Mediterranean flour moth, *Ephestia kuehniella* (*E. kuehniella*) Zeller (Lepidoptera: Pyralidae) [6,15,23,24]. Unfortunately, *E*. *kuehniella* eggs are expensive, limiting their usefulness as cost-effective food to mass-produce predators [25,26]. One possible solution is a species of brine shrimp, *Artemia franciscana* (*A. franciscana*) Kellogg (Anostraca: Artemiidae); the eggs (*i.e*., decapsulated cysts) of which are much less expensive to culture than *E*. *kuehniella* eggs [27]. Although cheaper to produce, the nutritional value or the capacity of *A*. *franciscana* eggs to support the growth, development and reproduction of predators needs further study. *C*. *maculata* were reared successfully on *A*. *franciscana* eggs in research in the recent past [13], but information on the effects of this food on development and reproduction were not reported.

In more recent work, *A*. *franciscana* eggs were found suitable for *C*. *maculata* growth and development, but not reproduction [15]. The crude protein in *A*. *franciscana* eggs was higher than in *E*. *kuehniella* eggs [15,26], the amino acid profiles were similar [26], but the soluble protein content was higher in *E*. *kuehniella* [15]. In addition, differences in egg hardness or texture (*A*. *franciscana* eggs are much harder than *E*. *kuehniella* eggs) could affect digestibility and assimilation of nutrients from *A*. *franciscana* eggs. Reducing the hardness, texture, and, maybe, particle size of *A*. *franciscana* eggs, via conversion into a powdered formulation, could render them more digestible by *C*. *maculata*. No previous work on the utilization of powdered *A*. *franciscana* eggs as a suitable food for development or reproduction of any arthropod has been reported in the literature.

Another possible reason for less than optimal reproduction could have been due to lower lipid (fatty acid) content in *A*. *franciscana* than in *E*. *kuehniella* eggs. In a previous study, palmitic (16:0), oleic (18:1), and linoleic (18:2) acids were more prevalent in *E*. *kuehniella* eggs than in *A*. *franciscana* eggs [26]. Fatty acid content (especially oleic acid and palmitic acid) is significantly greater in *E*. *kuehniella* eggs than in the pea aphid, *Acyrthosiphon pisum* (Harris) [23], a natural prey of *C*. *maculata*. Lipids (fatty acids) comprise a significant proportion of the dry weight of insect eggs, are an essential source of energy for developing embryos, and are transferred into the egg from the fat body or from the diet of the mother [28,29,30].

Palmitic acid (16:0) is one of the predominant free fatty acids in insects [31,32] and occurs in the tissues of coccinellids, even in *C*. *maculata* [33]. Palmitic acid along with hexanoic (6:0), myristic (14:0), sorbic (6:2), and stearic (18:0) acids are commonly found in fatty acid profiles in the tissues and cornicle secretions of aphid species [34,35,36,37]. Aphids are essential food for many coccinellids including *C*. *maculata* [7]. There has been meager research on the transfer of fatty acids from natural prey (e.g., aphids), factitious foods, or artificial diets to coccinellids, and how fatty acids affect life history parameters. There is limited evidence that the transfer of fatty acids from host plant to prey (aphids) improves the growth, development, and pre-imaginal survival of ladybird beetles, *C*. *maculata*, *Hippodamia convergens* Guérin-Méneville, *Coccinella septempunctata* L., and *Propylaea japonica* (Thunberg) [38,39,40]. When incorporated into yeast- and casein-based artificial diets, myristic and stearic acids enhanced growth, development and fecundity of the ladybird beetle *Olla abdominalis* (Say), syn., *v*-*nigrum* (Mulsant) [17,41]. Myrisitc and stearic acids could also function as precursors in the biosynthesis of defensive alkaloids commonly found in coccinellid tissues and blood [42].

More research is necessary to determine if incorporating palmitic acid into factitious foods (or artificial diets) can serve as an added source of energy to enhance development and reproduction (oviposition) in *C*. *maculata* and other ladybird species. In this study, we evaluated the effects of powdered *vs.* whole brine shrimp (*A*. *franciscana*) eggs supplemented with palmitic acid on life parameters of *C*. *maculata*. We tested the hypotheses that (1) powdered *A*. *franciscana* eggs are more suitable than whole eggs and (2) palmitic acid is an effective dietary supplement.

## 2. Experimental Section

### 2.1. Insect Colonies and Food Sources

Our *C*. *maculata* colony originated from adults provided by colleagues at USDA facilities in Brookings, SD and Beltsville, MD, USA. Each life stage (eggs, larvae, pupae or adults) was reared in separate containers in a climate-controlled room (24 °C, 50%–60% RH, 16 h photophase). This colony has been maintained without any introduction of “wild” individuals for over 20 consecutive generations. Larvae and adults were reared on *E*. *kuehniella* eggs. Frozen-fresh *E*. *kuehniella* eggs were purchased from Beneficial Insectary Inc. (Redding, CA, USA) at a cost of $ 3.80 USD per 28.35 g [15]. Upon arrival, eggs were stored in a lab refrigerator (at −20 °C), until ready for use.

### 2.2. Powdered *vs*. Whole Brine Shrimp Eggs with Palmitic Acid

To begin this experiment, *C*. *maculata* egg clutches were harvested at random from different rearing containers (from our colony in the climate-controlled room) housing 1-month-old mating pairs and setup in Petri dishes (clear plastic, 2.5 cm high, 9.0 cm wide, and 159 cm^3^ volume). Egg hatch typically began within 2–3 days and once 1st instars began moving on or away from the remnants of egg clutches, we removed them one at a time using a fine camel hair paint brush from the original Petri dishes and assigned them to clean, experimental Petri dish arenas (of the same size).

We used a Waring^®^ 1 L blender (catalog no. EF22302A, A. Daigger & Company Inc., Vernon Hills, IL, USA), set at 18,000 rpm for 1 min, then 22,000 rpm for 5 min per run, to convert “whole” (freeze-dried, decapsulated cysts) brine shrimp eggs, designated as whole BSE, into a dust-like, powdered formulation, designated as powder BSE. Note that the fine powder became airborne, during the blending procedure, and collected on the underside of the lid. This fine powder was harvested and placed in a clean plastic container, with an air-tight lid, then stored in a lab freezer (−20 °C) until ready for use.

We selected palmitic acid (product no. P0500, ≥99% purity, Sigma-Aldrich Corporation, St. Louis, MO, USA), because it was relatively inexpensive ($3.83 USD per g), readily available in a crystallized form that can be ground into a fine powder and easily combined with whole or powder BSE, using a mortar and pestle. After weighing (in mg), we added the palmitic acid powder with eggs using a mortar and pestle. The treatments in this experiment were formulated based on weight (mg/mg); 100% powder BSE, 95% powder BSE + 5% 16:0 (palmitic acid), 100% whole BSE, and 95% whole BSE + 5% 16:0. We limited the palmitic acid concentration to 5%, because 10 and 20% concentrations had harmful effects on *C*. *maculata* development and reproduction (Riddick, E.W. and Wu, Z., unpublished data [43]).

Each Petri dish arena (clear plastic, 2.5 cm high, 9.0 cm wide, and 159 cm^3^ volume) was provisioned with treatment food and covered with a mesh-screened lid. The quantity of treatment food in each arena (up to 40 mg, fresh weight) far exceeded the amount that *C*. *maculata*, collectively, could consume in two days. Treatment food was replaced, and uneaten food discarded, every other day. Each arena was provisioned with a small wad of cotton moistened with distilled water to provide a moisture source. Ten *C*. *maculata* 1st instars were randomly assigned to three replicate arenas in the four treatments; this experiment was replicated two times. Thus, *C*. *maculata* development was monitored in a total of 24 Petri dish arenas. Because 10 *C*. *maculata* 1st instars were initially in three replicate arenas in the four treatments, 120 were in each trial or 240 for the entire experiment (two trials combined). Since our overarching goal was time- and cost-effective mass production, we preferred experimental rearing of *C*. *maculata* in groups, rather than singly [15]. Collected data included development time, pre-imaginal survival, adult emergence rate, body size (live weight of teneral females and males) and sex ratio (reported as percentage of females emerging from pupae). To collect data on reproduction (pre-oviposition period, oviposition rate and egg hatch), each newly emerged female was paired with a male (reared on the same treatment food since 1st instar) in a clean Petri dish arena, and provided with the same food and water treatments since 1st instar. Males remained in arenas with females during the pre-oviposition period (the number of days before females began laying eggs). Arenas were checked twice daily for egg clutches, which were deposited on the walls of the arenas or on Kimwipes™ (A. Daigger & Co., Vernon Hills, IL, USA), strips at the base of arenas, and the total number of eggs (pooled across egg clutches) laid by each female was recorded. Egg hatch was also monitored each day.

All experimental arenas in both phases of this experiment (assessment of growth and development; assessment of reproduction) were maintained in a growth chamber (24 °C, 60% RH, 16 h photophase), but removed daily to record life history data, replenish food every other day and re-moisten cotton wads as needed. This experiment was halted on the 85th day from its initiation (*i.e*., when 1st instar *C*. *maculata* were added into arenas) in the two replicate trials.

### 2.3. Statistical Analysis

Data on development were averaged across replicate Petri dish arenas; the arena represented the sampling unit. Data on adult body weight and reproduction were averaged across adults; the adult represented the sampling unit. Initially, all data were analyzed following a completely randomized design and absolute data and percentage data were square-root transformed and arcsine transformed, respectively, if the assumptions of equal variance and normality were not met [44]. The one-way analysis of variance (one-way ANOVA), using the Standard Least Squares procedure with *F* statistic, tested the effects of food type on development, survival, sex ratio, and body weight of teneral males and females. Reproduction data (particularly, pre-oviposition time and progeny hatch rate) failed to meet the assumption of normality, even after transformation; the non-parametric Kruskal-Wallis analysis of variance (K-W ANOVA) with *H* statistic was used to test the effects of food type on pre-oviposition time, oviposition (total eggs laid per female), oviposition rate/day (eggs laid per day per female), and progeny (egg) hatch rate. A Spearman Rank Order Correlation analysis compared oviposition with pre-oviposition time, female body weight, and progeny hatch rate. A Simple Linear Regression analysis was used to calculate a regression line, when a significant correlation was detected. Treatment medians or means were significantly different following the K-W ANOVA or the one-way ANOVA, if *p* < 0.05. When necessary, the Steel-Dwass test or Tukey–Kramer HSD test was used as a multiple comparison procedure, after the K-W ANOVA or one-way ANOVA, respectively. JMP 10.0.0 (2012, SAS Institute Inc., Cary, NC, USA) and SigmaStat 3.0.1 interfaced through Sigma Plot 12 (Systat Software Inc., Richmond, CA, USA) software assisted with analysis of data.

## 3. Results

Powdered or whole brine shrimp eggs, with or without 5% 16:0 (palmitic acid), had no differential effect on the percentage of *C*. *maculata* surviving the larval stage (*F* = 1.83; df = 3, 20; *p* = 0.17) and all pupae survived to the adult stage, regardless of food treatment (Table 1). Time required for larvae to develop into pupae (*F* = 2.90; df = 3, 20; *p* = 0.06), then pupae into teneral adults (*F* = 0.89; df = 3, 20; *p* = 0.46) was not affected by food treatments. Sex ratio (percentage females) of teneral adults was unaffected as well (*F* = 0.51; df = 3, 20; *p* = 0.67; Table 1). A total of 92 females and 70 males emerged in the two trials combined. Food treatments had no effect on body weight of teneral males (*F* = 1.20; df = 3, 66; *p* = 0.32) or teneral females (*F* = 0.29; df = 3, 88; *p* = 0.83).

Food treatments had no differential effects on pre-oviposition time (*H* = 2.20; df = 3; *p* = 0.53; *n* = 63 females; K-W ANOVA). The median values with 25% and 75% confidence intervals for pre-oviposition time, oviposition (total eggs laid), oviposition rate/day (eggs laid per day), and hatch rate are indicated in Table 2. Note that pre-oviposition time was typically between 7–30 days after emergence for most females, regardless of food treatment; pre-oviposition time extended beyond 40 days for a few females fed whole eggs, with or without 5% palmitic acid. Total oviposition was greater for females fed powdered eggs than whole eggs and powdered eggs with 5% palmitic acid than whole eggs with or without 5% palmitic acid (*H* = 23.20; df = 3; *p* < 0.001; Table 2). The median total oviposition per female fed powdered eggs was two-fold greater than those fed whole eggs; median total oviposition per female fed powdered eggs with 5% palmitic acid was four-fold greater than those fed whole eggs (see Table 2). However, the oviposition rate per day did not differ significantly between treatments (*H* = 1.41; df = 3; *p* = 0.70).

**Table 1:** Mean ± SE pre-imaginal survival, development time, and sex ratio of *C*. *maculata* fed four different food treatments.

Parameter	Powder BSE + 5% 16:0	Whole BSE + 5% 16:0	Powder BSE	Whole BSE
Larval Survival (%)	66.67 ± 6.15 ^a^	65.00 ± 5.00 ^a^	76.67 ± 3.33 ^a^	63.33 ± 2.11 ^a^
Time as Larva (days)	13.08 ± 0.29 ^a^	13.54 ± 0.33 ^a^	13.50 ± 0.27 ^a^	14.25 ± 0.24 ^a^
Time as Pupa (days)	3.98 ± 0.13 ^a^	3.99 ± 0.22 ^a^	4.07 ± 0.08 ^a^	3.75 ± 0.12 ^a^
Pupal Survival (%)	100	100	100	100
Sex Ratio (% females)	54.52 ± 3.83 ^a^	53.85 ± 5.25 ^a^	56.00 ± 7.22 ^a^	62.72 ± 5.85 ^a^
Male Body Weight (mg)	11.71 ± 0.18 ^a^	11.51 ± 0.26 ^a^	11.82 ± 0.28 ^a^	11.01 ± 0.52 ^a^
Female Body Weight (mg)	13.58 ± 0.36 ^a^	13.67 ± 0.20 ^a^	13.75 ± 0.21 ^a^	13.42 ± 0.28 ^a^

BSE represents brine shrimp eggs; 5% 16:0 represents 5% palmitic acid. Mean ± SE values followed by the same letter in a row are not significantly different (*p* > 0.05). Sample size (n) was six arenas per treatment for preimaginal survival, development time, and sex ratio. For body weight, sample size (n) was 18, 18, 20, and 14 teneral males and 22, 21, 25, and 24 teneral females in powder BSE + 5% 16:0, whole BSE + 5% 16:0, powder BSE, and whole BSE treatments, respectively.

**Table 2:** Median (with 25% and 75% confidence intervals) pre-oviposition time, total oviposition, daily oviposition, and progeny hatch rate of *C*. *maculata* fed four different food treatments.

Parameter	Powder BSE + 5% 16:0	Whole BSE + 5% 16:0	Powder BSE	Whole BSE
Pre-oviposition Time (days)	14.0 ^a^ (10.0; 25.0)	14.5 ^a^ (10.0; 26.0)	12.0 ^a^ (10.0; 18.0)	15.0 ^a^ (12.0; 31.0)
Oviposition (eggs per female *)	69.0 ^a^ (30.0; 122.0)	23.0 ^bc^ (15.2; 47.5)	33.0 ^ab^ (28.0; 63.0)	15.0 ^c^ (10.5; 25.5)
Oviposition Rate/Day (eggs per female/day)	15.0 ^a^ (12.0; 18.0)	14.75 ^a^ (9.9; 19.5)	13.3 ^a^ (12.0; 16.8)	13.0 ^a^ (8.4; 19.0)
Hatch Rate (%)	67.2 ^a^ (60.0; 77.8)	62.8 ^a^ (58.3; 72.4)	68.0 ^a^ (62.4; 74.6)	52.9 ^a^ (43.3; 65.6)

* Eggs laid within a specific time frame (described in “Experimental Section” of manuscript). Median values followed by the same letter in a row (for separate parameters) are not significantly different (*p* > 0.05). Sample size (n, females) indicated for each treatment: powder BSE + 5% 16:0 (15), whole BSE + 5% 16:0 (16), powder BSE (19), and whole BSE (13).

**Figure 1:**
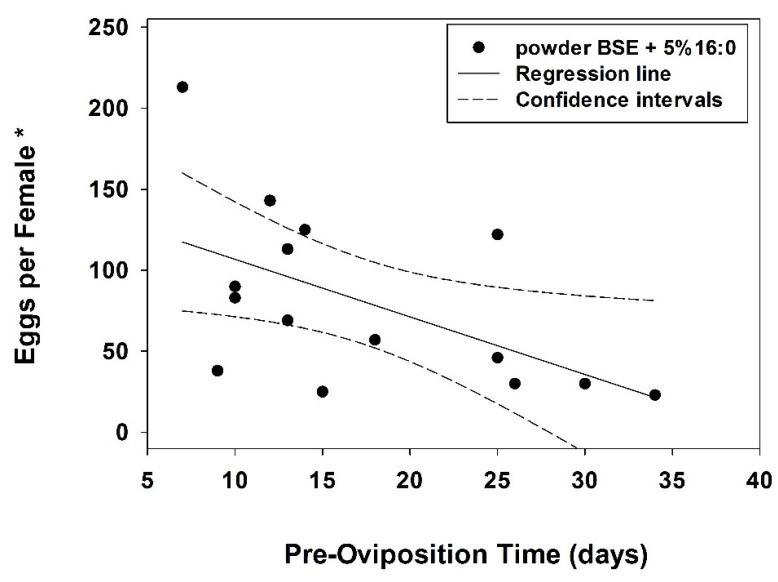
Regression between pre-oviposition time *vs.* total oviposition (number of eggs laid) per *C*. *maculata* female fed powdered BSE + 5% 16:0 (sample size, 15 females). The regression line defines the relationship between pre-oviposition time (*x*) and eggs laid per female (*y*). Simple linear regression; *y* = *y*_o_ + a*x*; *y*_o_, 142.28; a, −3.55; *r*^2^ = 0.303; adjusted *r*^2^ = 0.250 (*n*, 15 observations). * Eggs laid within a specific time frame (described in “Experimental Section” of manuscript).

There was a negative correlation between oviposition (total number of eggs laid) and pre-oviposition time for females fed powdered eggs with 5% palmitic acid (*r*_s_ = −0.597; *p* = 0.018; *n* = 15 females), but not for females fed whole eggs with 5% palmitic acid (*r*_s_ = −0.24; *p* = 0.37; *n* = 16), powdered eggs alone (*r*_s_ = −0.10; *p* = 0.69, *n* = 19) or whole eggs alone (*r*_s_ = −0.05; *p* = 0.86; *n* = 13). A weak functional relationship existed between oviposition and pre-oviposition time for females fed powdered eggs with 5% palmitic acid; oviposition increased as pre-oviposition time decreased (*F* = 5.66; df = 1,13; *p* = 0.03; *r*^2^ = 0.303; Figure 1). Approximately 50% of females fed powdered eggs with 5% palmitic acid began oviposition 20 days after emergence as adults; 100% of them began in 35 days (Figure 1). Oviposition was not correlated with body weight or with progeny (egg) hatch rate of females amongst food treatments (statistics not shown) and hatch rate did not differ significantly amongst food treatments (*H* = 7.04; df = 3; *p* = 0.071; see Table 2).

## 4. Discussion

Our hypothesis that powdered eggs are more suitable than whole eggs was partially confirmed, since *C*. *maculata* oviposition, *i.e*., total eggs laid by females within the timeframe of the experiment, was improved. The two-fold increase in median oviposition of females fed *A*. *franciscana* powdered eggs alone, in comparison to whole eggs, confirms the superiority of powdered eggs. In a recent study, whole *A*. *franciscana* eggs were not suitable for *C*. *maculata* reproduction, when compared against *E*. *kuehniella* eggs [15]. The reasons why *A*. *franciscana* powdered eggs are more suitable are unclear. Perhaps, altering the hardness, texture, or particle size of eggs, *i.e*., conversion from whole to powder form, renders them easier to digest or assimilate, therefore inducing a greater oviposition rate. Conversely, factors other than ease of digestion and assimilation must be involved in the changes seen in oviposition, because the consumption of powdered eggs did not signal significant improvements in pre-imaginal survival, growth rate, and development of *C*. *maculata*. Notwithstanding, we know that nutritional needs of larvae and adults can be slightly different [6]. No comparable research to help explain the superiority of *A*. *franciscana* powdered eggs over whole eggs as a food source that boosts reproduction in *C*. *maculata*, or any other ladybird beetle, has been reported previously to our knowledge.

Previous work does, however, demonstrate that *C*. *maculata* and another ladybird, *Anatis mali* (Say), can develop to the adult stage on animal-based foods of a powder-like consistency. For example, they complete their development on dried, powdered nymphs and adults of the corn leaf aphid *Rhopalosiphum maidis* (Fitch) or pea aphid *Acyrthosiphon pisum* (Harris) [8,9]. Whether or not powdered aphids are more suitable than whole (live or dried) aphids for *C*. *maculata* reproduction, or reproduction of any other ladybird species, is unclear.

Our hypothesis that 5% palmitic acid is an effective supplement was partially confirmed, since adding it to powdered *A*. *franciscana* eggs appeared to improve reproduction (oviposition) two-fold over powdered eggs alone (see Table 2). Perhaps, 5% palmitic acid hastened the onset of oviposition in some females. A follow-up experiment could involve dissecting females to determine ovarian development after being fed a diet of powdered *A*. *franciscana* eggs with or without 5% palmitic acid. We predict that egg maturation will be more advanced, by at least several days, in the ovaries of females fed powdered eggs with 5% palmitic acid than powdered eggs alone.

It is generally assumed that coccinellids emerge with very few or no mature eggs in their ovaries, *i.e*., synovigenic [45] and require at least several days to mature eggs before any viable ones are laid. *C*. *maculata* reportedly possess 14 ovarioles in mature ovaries [46], presumably signaling their potential of generating many eggs over time, but information on oogenesis (egg maturation) is unavailable. The timeframe of the entire experiment—rom inception to termination—was 85 days. This meant that the time allotted to oviposition could be brief, a few weeks, to moderate, up to two months, depending on the pre-oviposition time. This explains, in part, why females fed powdered *A*. *franciscana* eggs with 5% palmitic acid showed an increase in oviposition with a decrease in pre-oviposition time; they had more time (days) to oviposit. The observation that the number of eggs laid per day (*i.e*., per capita oviposition) did not differ amongst the treatments further supports our contention that some of the females fed powdered eggs with 5% palmitic acid had more time to lay eggs. Also, the observation that progeny (egg) hatch rate did not decline significantly when females were fed powdered eggs (with or without 5% palmitic acid) rather than whole eggs demonstrates that egg viability was not affected. There is no comparable published research on the utilization of palmitic acid as a supplement to *A*. *franciscana* eggs (powdered or whole forms) or any other alternative food, in lieu of natural prey (e.g., aphids).

The capacity of fatty acids or lipids, in general, to enhance oviposition in ladybird beetles, whether used as a supplement to a food source, or as a standalone resource, has been shown before. For example, incorporating myristic and stearic acids into an artificial diet enhanced the fecundity of the ladybird *O*. *abdominalis* [17,41]. There is also evidence that myristic acid is potentially transferred from alfalfa plants to pea aphids, *A*. *pisum*, then to its predators, *C*. *maculata* and *H*. *convergens*, to reduce development time and increase pre-imaginal survival [38], but the effects on oviposition are not reported. The free fatty acid concentration in cotton cultivar M9101, which contained a high gossypol content, was transferred to the cotton aphid *Aphis gossypii* Glover, then to its predator, *P*. *japonica*, to improve growth and development [40]; effects on oviposition were not reported. Further research is necessary to demonstrate the value of supplementing alternative foods (and artificial diets) with fatty acids to enhance development as well as reproduction in more coccinellid species.

## 5. Conclusions

This study represents an attempt at boosting the nutritional value of the more cost-effective *A*. *franciscana* eggs to support the mass production of *C*. *maculata*. Although we demonstrate that powdered *A*. *franciscana* eggs increase oviposition, follow-up research is required to prove, convincingly, that 5% palmitic acid hastens the onset of oviposition. This may involve dissecting females to gauge their ovarian development, *i.e*., egg maturation rate, when feeding continuously on powdered eggs alone or powdered eggs with 5% palmitic acid.

The ultimate goal of our research is to increase the suitability of alternative foods to support cost-effective, large-scale rearing of *C*. *maculata* and other ladybirds for augmentative biological control. The next logical step is to demonstrate that powdered eggs, supplemented with fatty acids, can hasten the reproduction of *C*. *maculata* on a large enough scale to support inundative releases in high tunnels, greenhouses and plantscapes.
